# A cross-sectional study on quality of life among the elderly in non-governmental organizations’ elderly homes in Kuala Lumpur

**DOI:** 10.1186/s12955-016-0408-8

**Published:** 2016-01-12

**Authors:** Obinna Francis Onunkwor, Sami Abdo Radman Al-Dubai, Philip Parikial George, John Arokiasamy, Hemetram Yadav, Ankur Barua, Hassana Ojonuba Shuaibu

**Affiliations:** Department of Community Medicine, International Medical University (IMU), Kuala Lumpur, Malaysia; Faculty of Medicine, SEGi University, Kota Damansara, Selangor Malaysia

**Keywords:** Elderly, Non-governmental Organization, Elderly Homes, Quality of Life, Kuala Lumpur

## Abstract

**Background:**

There is a rapid increase in the population of the elderly globally, and Malaysia is anticipated to become an ageing nation in 2030. Maintaining health, social participation, reducing institutionalization, and improving quality of life of the elderly are public health challenges of the 21^st^ century. Quality of life among elderly in Elderly Homes in Malaysia is under researched. This study aims to determine the quality of life and its associated factors among the Elderly in Elderly Homes in Kuala Lumpur.

**Methods:**

This was a cross-sectional study among 203 residents aged 60 years or more in eight randomly selected Elderly Homes in Kuala Lumpur in September 2014. Stratified simple random sampling was used to select participants. Study instruments included World Health Organization Quality of Life Questionnaire-Brief Version (WHOQOL-BREF), Multidimensional Scale for Perceived Social Support, and a questionnaire for Socio-demographic variables. Data collection was by face to face interview. Univariate and Multivariate analysis were used to determine associations, and *P*-value <0.05 was considered statistically significant.

**Results:**

The mean (Standard deviation) for the physical domain was 14.3 (±2.7), 13.7 (±2.5) for the psychological domain, 10.8 (±3.4) for the social domain, and 13.0 (±2.5) for the environment domain. Factors significantly associated with quality of life included age, gender, level of education, economic status, outdoor leisure activity, physical activity, duration of residence, type of accommodation, co-morbidities, and social support.

**Conclusion:**

This study confirms that multiple factors are associated with quality of life among elderly in elderly homes. Social support, chronic co-morbidities, gender and outdoor leisure activity were significantly associated with all the domains of quality of life. Among the four domains of quality of life, the physical domain had the highest score while the social domain had the lowest score. This emphasizes the need for more social support-related interventions in these homes.

## Background

Presently the population of people aged 60 years and above is increasing rapidly in the world. This phenomenon is attributed to longer life expectancy, low fertility rates, remarkable public health policies, and advances in medicine and health care [1, 2].

In Malaysia, the elderly constituted about 8.2 % (2.4 million) out of a total population of 30 million people in 2012, and they will make up over 15 % of the population by 2030 [3, 4]. Studies about quality of life among the elderly are essential because they evaluate the efficacy of health intervention, welfare programs, health care and well-being of the elderly. The World Health Organization (WHO) defined quality of life (QOL) as an individual’s perception of his/her status in life in the context of the individual’s environment, belief systems and goals [5]. It is an indicator for active ageing. Active ageing is the process of optimizing health, and enhancing quality of life of the elderly. It is at present on the fore as an objective for the ageing population [6, 7].

Previous studies have linked social support to quality of life. Social support is real resources or perceived resources offered to others to enable them feel valued [8]. It can be vital for elderly who depend on organizations or friends for routine activities, companionship, and to provide the needed care for their well-being [9]. Studies have shown that positive social relationship (with family, friends, and neighbors) promotes quality of life. In contrast, decreased social contacts which could occur through loss of members of a social network is significantly associated with poor quality of life [10, 11]. Higher levels of social support has been linked to reduced risk of mental disorders, diseases, mortality and improved quality of life [12–14]. Studies conducted in developing countries have reported that the quality of life of institutionalized elderly is poorer than that of community-dwelling elderly [15, 16]. Quality of life among the elderly in elderly homes in Malaysia is under researched, as most of such studies focus on community-dwelling elderly. There were over 6000 elderly residing in elderly homes in 2012 in Malaysia and the number is anticipated to rise [17]. These non-governmental organizations’ elderly homes are unique institutions in Malaysia. They do not provide nursing services and residents are mostly elderly people that were abandoned in hospitals by their families. These residents are faced with challenges ranging from poor access to health care, decline in social participation, neglect by family and friends, and unfriendly interactions such as reprimands and disturbances during sleep, all of which could affect quality of life. Maintaining health of the elderly, autonomy for as long as possible, increasing social participation, dignity, reducing institutionalization and improving quality of life have become immense public health challenges in the 21^st^ century, and as the number of elderly in elderly homes increases across Malaysia, becoming aware of their quality of life and factors that influence it also grows in importance. This study aims to assess the quality of life among the elderly in elderly homes, and determine factors associated with the quality of life, such as social support, type of accommodation, duration of residence in the home, among others.

## Methods

### Design, setting, and sampling

This cross-sectional study was conducted in eight elderly homes in Kuala Lumpur between September to November 2014. These non-profit homes are run by non-governmental organizations. Residents of these homes are mostly elderly citizens abandoned in hospitals in Kuala Lumpur. Other residents are neglected or poor elderly citizens without a shelter. Residents are referred to these homes by the hospitals, family members, neighbors, and sometimes by the Department of Social Welfare Malaysia.

### Facilities and services in the elderly homes

Unlike nursing homes and retirement homes that charge monthly residential fees, these homes provide residential services without a fee. They rely enormously on philanthropy of the public and they do not provide nursing services. Hence a basic criteria for admission into these homes is the ability to perform activities of daily living. These elderly homes provided accommodation in either the ward-type accommodation or the twin-sharing accommodation. The twin-sharing accommodation had two people (two beds) in a room, the ward-type accommodation had over two people (over two beds) in a room. In addition, unlike the ward-type, residents in the twin-sharing accommodation can close their door when they need privacy, and they can easily control who goes in and out of their room. On the other hand, the ward-type accommodation was like a large hall with several residents. The size of the homes varied from 16 to 59 beds. The number of staff also varied from 4 to 10. These homes had shared toilets and bathrooms, a kitchen, common dining area for meals, a gathering place for recreational and leisure activities like watching TV, playing cards, listening to music among others. These homes organize routine indoor exercise sessions in the morning and some are fitted with exercise equipment with the stationary bicycle the most common. Medical care is usually provided by volunteers who visit occasionally and are most times limited to physical examination without radiological and laboratory investigations. However these homes when they can afford it endeavor to take residents to hospitals. Occasionally outdoor activities are organized by the homes or volunteers and could include visit to a park, place of worship, or to a local theatre to see a movie. The homes lacked sufficient space for gardens, and wardrobes and libraries were also absent.

### Sample size estimation and sampling technique

Using a standard deviation of 10 for quality of life scores in elderly population, margin of error as 1.5, and a confidence level of 95 %, the minimum sample size for this study was determined to be 170. The total sample size became 203 after adding a non-response rate of 19 %. During the post study survey 13 elderly homes were identified in Kuala Lumpur. A spot map was used to identify the geographical locations of the homes. The homes were numbered serially on the map in a clockwise fashion beginning from the home farther north. Using the lottery method, simple random sampling was used to select eight homes. Eight homes were selected because it was more than half of the identified elderly homes, thus would be a good representation of the target population, and also because the number of residents in the eight homes was up to the estimated sample size. A total of 203 residents were selected by stratified (proportionate) random sampling. Firstly the proportion of participants to be selected from each home was determined by dividing the number of residents in that home by the total number of residents in the eight homes, then multiplying the fraction by 100. Secondly the total number of participants to be selected from each home was determined by dividing the proportion of residents to be selected in each home (obtained from the first step) by 100, then multiplying the fraction by the estimated sample size (203). Participants in each home were then selected randomly from a sampling frame of each home using the lottery method. The inclusion criteria included those aged 60 years and above, those who were able to understand English, Malay, or Chinese, and those who gave written consent to participate. Residents aged less than 60 years, unable to communicate, and those with cognitive impairment as indicated by their files in the homes were excluded from the study. Retirement homes, private homes, and homes that provided nursing services were not included in this study.

### Study instruments

A structured close-ended questionnaire was used in this study. The first part included questions on socio-demographic variables such as; age, gender, ethnicity, religion, marital status, number of children, level of education, previous employment sector, pension, socio-economic status, outdoor leisure activity, smoking status, alcohol status, physical activity, duration of stay in home, type of accommodation, co-morbidities, and self-rated health status. Outdoor leisure activity was defined as leisure activities outside the home such as a visit to a park, movie theatre, among others at least twice a month excluding visit to health centers for medical treatment. Physical activity was defined as 150 minutes of moderate intensity exercise or at least 75 minutes of vigorous- intensity exercise weekly [18], for type of accommodation, two types of accommodation were available; the twin-sharing accommodation had two people (two beds) in a room while the ward-type accommodation had over two people (over two beds) in a room. For self-rated health, participants were asked whether they perceived their general health as good or poor. Economic status was determined by asking participants to rate their current economic status as good, intermediate or poor. This method was used since participants were currently unemployed. It has been used in a previous study [19], and it is known to be a reliable means of obtaining such information as people can correctly grade their economic status with reference to standard of living in their community and compare themselves with others.

Quality of life was measured with the validated World Health Organization Quality of Life Instrument-Brief Version (WHOQOL-BREF). It evaluates perceived quality of life using 26 items categorised into Physical domain (7 items), Psychological domain (6 items), Social Relations domain (3 items), and Environment domain (8 items). Two items evaluates perception of general health and quality of life. Each item is ranked on a 5-point Likert scale. Higher scores indicate higher quality of life [5]. The English version of the instrument was validated in a previous study [20] and was found to have Cronbach’s alpha ranging from 0.68 to 0.82 across the four domains. The instrument also demonstrated good validity. The Malay version was validated in a previous study [21], and the Cronbach’s alpha ranged from 0.64 to 0.80 across the four domains. The Chinese version of the instrument have also been validated in a previous study [22], the Cronbach’s alpha ranged from 0.67 to 0.78 across the four domains. The multidimensional Scale for perceived social support (MSPSS) was used to measure social support. This instrument was developed and validated by Zimet et al. in 1988 [23]. It measures perceived availability of support. It has 12 items, and it measures social support from three sources of support: Family, Friends and Significant others (special person). Each source of support has 4 items. Each item is ranked on a 7-point Likert –Scale from 1: very strongly disagree to 7: very strongly agree. On the MSPSS, higher scores indicate higher level of social support. A total score of 69–84 indicate high level of perceived social support, 49–68, and 12–48 indicate moderate and low levels of social support respectively [24]. The English version was validated in previous studies [23, 25] and was found to have Cronbach’s alpha of up to 0.92. The instrument also demonstrated good validity. The Malay version was validated in a previous study [26], and was found to have a Cronbach’s alpha of 0.89. The Chinese version was validated in a previous study [27] and it demonstrated good psychometric properties with Cronbach’s alpha of 0.92 and test retest reliability of 0.71. All language versions of study instruments used in this study have demonstrated good psychometric properties. Data collection was by face to face interview. The questions were administered in Chinese, Malay and English languages by a trained native speaker of each language. A pilot study was conducted before the actual study commenced.

### Ethical consideration

The research and ethical committee of International Medical University Malaysia approved this research in 2014 (Project Identification Number; MScPHI1/2014(09). Permission was obtained from the administrators of each home to access the homes. All participants were given detailed information about the study objectives and confidentiality of the information, and a written consent was signed by each participant.

### Data analysis

Statistical Package for Social Sciences (SPSS) version 20.0 for windows was used to analyze the collected data. The results of continuous variables were expressed as means and standard deviations, while categorical variables were expressed as proportions and frequencies. Some continuous variables were dichotomized; social support was dichotomized into low level of social support and medium/high levels of social support, marital status was dichotomized into married and single (unmarried, separated, and widowed), level of education was dichotomized into none/primary and secondary/tertiary education.

T-test was used for univariate analysis, and only variables with *P*-value less than 0.05 was added to the multiple linear regression model. This model was used to identify the predictors of quality of life and *P*-value less than 0.05 was considered statistically significant.

## Results

### Characteristic of respondents

The age of the study participants ranged from 60 to 95. As shown in Table 1, of the 203 participants, 64.5 % were males and 35.5 % were females, 46.3 % were aged 60 to 69 years, and 53.7 % were aged 70 years or more. The mean age was 71.5 (±6.8). Majority of the respondents were Chinese (87.1 %). Most of the respondents were married (61.6 %) and 38.4 % were single, 57.6 % of the respondents had 1 child and 42.4 % had 2 children or more. Majority of the respondents had secondary education (46.8 %), while 39.9 % had primary or no education. Most of the respondents previously worked in the private sector (61.1 %), while 29.5 % were self-employed, and 3 % were unemployed. Most of the respondents were not receiving pension (90.6 %). Only 6.4 % perceived their economic status as good, 8.4 % reported an intermediate, and 85.2 % reported poor economic status. Most of the respondents engaged in outdoor leisure activities (78.3 %), and 31.5 % were physically active. Smoking and alcohol drinking was reported by 23.6 % and 7.9 % of respondents respectively. Majority (54.2 %) had lived in an elderly home for less than 2 years. Most of the respondents lived in ward-type accommodation (90.1 %) and 9.9 % lived in twin-sharing accommodation. Majority (82.8 %) had at least one chronic co-morbidity. Only 5.4 % reported high level of social support, while low and medium levels of social support was reported by 47.8 % and 46.8 % respectively.

**Table 1:** Characteristics of participants

Variable	*n*	%
Age		
60–69 years	94	46.3
≥70 years	109	53.7
Gender		
Female	72	35.5
Male	131	64.5
Ethnicity		
Malay	6	3.0
India	17	8.4
Chinese	177	87.1
Others	3	1.5
Marital status		
Married	125	61.6
Single	78	42.4
Number of children		
1 child	117	57.6
≥ 2	86	42.4
Level of education		
None	36	17.7
Primary	45	22.2
Secondary	95	46.8
Tertiary	27	13.3
Previous employment sector		
Government	13	6.4
Private	124	61.1
Self-employed	60	29.5
Unemployed	6	3.0
Pension		
Yes	19	9.4
No	184	90.4
Economic status		
Poor	173	85.2
Intermediate	17	8.4
Good	13	6.4
Outdoor leisure activity		
Yes	159	78.3
No	44	21.7
Smoking status		
Smoker	48	23.6
Non-smoker	155	76.4
Alcohol status		
Drinker	16	7.9
Non-drinker	187	92.1
Physical activity		
Yes	64	31.5
No	139	68.5
Duration of residence in home		
< 2 years	110	54.2
≥ 2 years	93	45.8
Type of accommodation		
Ward	183	90.1
Twin-sharing	20	9.9
Chronic co-morbidities		
Yes	168	82.8
No	35	17.2
Social support		
Low	97	47.8
Medium	95	46.8
High	11	5.4

### Univariate analysis

Table 2 shows the mean scores for the quality of life domains. The mean (SD) for the physical domain was 14.3 (±2.7), 13.7 (±2.5) for the psychological domain, 10.8 (±3.4) for the social domain, and 13.0 (±2.5) for the environment domain.

**Table 2:** Mean Scores for QOL Domains

Domain	Mean	SD (±)	Minimum	Maximum
Physical	14.3	2.7	6	20
Psychological	13.7	2.5	7	19
Social	10.8	3.4	4	20
Environment	13.0	2.5	8	19

The univariate analysis in Table 3 shows that among the socio-demographic variables, gender was significantly associated with all domains of QOL (*p* < 0.05). Age was significantly associated with the physical domain (*p* = 0.010). Level of education was significantly associated with the physical domain (*p* = 0.006), psychological domain (*p* = 0.009) and social domain (*p* = 0.007). Economic status was significantly associated with the physical domain (*p* = 0.037), psychological domain (*p* = 0.008), and social domain (*p* = 0.019). Duration of residence was significantly associated with psychological domain (*p* = 0.001), social domain (*p* = <0.0001) and environment domain (*p* = <0.0001). Type of accommodation was significantly associated the psychological domain (*p* = 0.001), social domain (*p* = 0.081), and environment domain (*p* = 0.004).

**Table 3:** Factors associated with QOL Domains in Univariate Analysis

Variable	Physical	Psychological	Social	Environment
	Mean( ±)	Mean( ±)	Mean( ±)	Mean( ±)
Age				
60–69 years	14.8 (2.6)	13.7 (2.4)	10.9 (3.5)	12.8 (2.4
≥ 70 years	13.8 (2.7)	13.6 (2.6)	10.7 (3.4)	12.9 (2.7)
*P-value*	*0.010*	*0.890*	*0.752*	*0.806*
Gender				
Female	12.9 (2.9)	12.7 (2.5)	9.5 (3.5)	12.2 (2.5)
Male	15.1 (2.3)	14.2 (2.4)	11.4 (3.2)	13.3 (2.5)
*P-value*	*<0.0001*	*<0.0001*	*<0.0001*	*0.003*
Ethnicity				
India	14.2 (3.3	14.1 (1.9)	10.4 (2.9)	13.1 (1.7)
Malay	13.1 (3.9)	14.3 (2.7)	13.2 (4.4)	13.3 (3.1)
Chinese	14.4 (2.6)	13.6 (2.5)	10.7 (3.4)	12.9 (2.6)
Others	14.0 (3.6)	13.7 (4.1)	12.0 (8.0)	13.7 (2.5)
*P-value*	*0.766*	*0.782*	*0.309*	*0.926*
Marital status				
Single	14.4 (2.8)	13.3 (2.4)	10.4 (3.2)	13.2 (2.6)
Married	14.2 (2.6)	13.8 (2.5)	10.9 (3.5)	12.7 (2.5)
*P-value*	*0.645*	*0.155*	*0.256*	*0.211*
Level of education				
None/primary	13.6 (2.9)	13.1 (2.6)	9.9 (3.2)	12.6 (2.6)
Secondary/tertiary	14.7 (2.5)	14.0 (2.3)	11.2 (3.4)	13.1 (2.4)
*P-value*	*0.006*	*0.009*	*0.007*	*0.194*
Pension				
Yes	14.1 (3.1)	14.3 (3.1)	10.5 (3.3)	12.7 (3.1)
No	14.3 (2.7)	13.6 (2.4)	10.8 (3.4)	12.9 (2.5)
*P-value*	*0.668*	*0.238*	*0.748*	*0.662*
Economic status				
Poor/intermediate	14.2 (2.6)	13.5 (2.4)	10.6 (3.3)	12.8 (2.5)
Good	15.8 (3.1)	15.4 (2.8)	12.9 (3.7)	13.8 (3.1)
*P-value*	*0.037*	*0.008*	*0.019*	*0.187*
Outdoor leisure activity				
Yes	14.8 (2.4)	13.9 (2.4)	11.2 (3.4)	13.1 (2.5)
No	12.7 (3.0)	12.6 (2.5)	9.3 (2.8)	12.1 (2.6)
*P-value*	*<0.0001*	*0.002*	*0.001*	*0.021*
Smoking status				
Smoker	14.1 (2.4)	13.3 (2.4)	10.3 (3.1)	12.9 (2.6)
Non-smoker	14.4 (2.8)	13.8 (2.5)	10.9 (3.5)	12.8 (2.5)
*P-value*	*0.475*	*0.216*	*0.233*	*0.984*
Alcohol status				
Drinkers	14.1 (2.5)	13.6 (2.5)	10.1 (3.6)	13.1 (2.6)
Non-drinker	14.3 (2.8)	13.7 (2.5)	10.8 (3.4)	12.9 (2.6)
*P-value*	*0.779*	*0.866*	*0.394*	*0.755*
Physical activity				
yes	15.0 (3.1)	14.1 (2.6)	11.6 (3.2)	13.5 (2.6)
No	13.9 (2.5)	13.5 (2.4)	10.4 (3.4)	12.6 (2.5)
*P-value*	*0.013*	*0.078*	*0.025*	*0.021*
Duration of residence in home				
< 2 years	14.0 (2.8)	13.1 (2.3)	9.6 93.1)	12.3 (2.5)
≥ 2 years	14.6 (2.6)	14.3 (2.5)	12.1 (3.4)	13.6 (2.4)
*P-value*	*0.135*	*0.001*	*<0.0001*	*<0.0001*
Type of accommodation				
Ward	14.3 (2.8)	13.6 (2.5)	10.5 (3.3)	12.7 (2.5)
Twin-sharing	14.1 (2.7)	14.6 (2.4)	13.2 (3.5)	14.5 (2.1)
*P-value*	*0.656*	*0.001*	*0.081*	*0.004*
Chronic co-morbidities				
Yes	13.9 (2.5)	13.3 (2.4)	10.2 (3.2)	12.6 (2.6)
No	16.2 (2.9)	15.5 (2.4)	13.5 (3.2)	14.6 (1.8)
*P-value*	*<0.0001*	*<0.0001*	*<0.0001*	*<0.0001*
Social support				
Low	13.4 (2.7)	12.1 (2.1)	8.6 (2.7)	11.5 (2.3)
High/medium	15.1 (2.5)	15.1 (1.9)	12.7 (2.8)	14.3 (2.1)
*P-value*	*<0.0001*	*<0.0001*	*<0.0001*	*<0.0001*

Independent t-test was used for analysis and *p*-value <0.05 was considered statistically significant

Outdoor leisure activity, chronic co-morbidity, and social support were significantly associated with all domains of QOL (*p* = <0.05). Pension, ethnicity marital status, smoking status, and alcohol status were not significantly associated with any domain of quality of life.

### Multivariate analysis

Tables 4, 5, 6 and 7 show the results of the multivariate analysis. Variables significantly associated with the physical domain of quality of life included; age (*p* = 0.021), gender (*p* = <0.0001), economic status (*p* = 0.037), outdoor leisure activity (*p* = <0.0001), chronic co-morbidities (*p* = 0.020) and social support (*p* = 0.013).

**Table 4:** Factors associated with Physical Domain of QOL in Multivariate Analysis

Variable	B	S.E.	Beta	t	P-value	95 % C.I	
						Lower	Upper
Age							
60–69 years	ref	ref	ref	ref	ref	ref	ref
≥ 70 years	-0.77	0.33	-0.14	-2.32	0.021	-1.42	-0.11
Gender							
Female	ref	ref	ref	ref	ref	ref	ref
Male	1.71	0.34	0.29	4.95	<0.0001	1.02	2.39
Level of education							
None/primary	ref	ref	ref	ref	ref	ref	ref
Secondary/tertiary	0.46	0.33	0.08	1.38	0.167	-0.19	1.12
Economic status							
Poor	ref	ref	ref	ref	ref	ref	ref
Good	1.42	0.67	0.12	2.10	0.037	0.08	2.76
Outdoor leisure activity							
Yes	ref	ref	ref	ref	ref	ref	ref
No	1.43	0.39	0.21	3.62	<0.0001	0.65	2.21
Physical activity							
Yes	ref	ref	ref	ref	ref	ref	ref
No	0.49	0.36	0.08	1.37	0.171	-0.21	1.21
Chronic co-morbidities							
Yes	ref	ref	ref	ref	ref	ref	ref
No	1.10	0.47	0.15	2.35	0.020	0.17	2.04
Social support							
Low	ref	ref	ref	ref	ref	ref	ref
High/medium	0.64	0.34	0.15	2.50	0.0013	0.18	1.54

Multiple linear regression was used for analysis and *p*-value <0.05 was considered statistically significant

**Table 5:** Factors associated with Psychological Domain of QOL in Multivariate Analysis

Variable	B	S.E.	Beta	t	P-value	95 % C.I	
						Lower	Upper
Gender							
Female	ref	ref	ref	ref	ref	ref	ref
Male	0.88	0.29	0.16	3.05	0.003	0.31	1.46
Level of education							
None/primary	ref	ref	ref	ref	ref	ref	ref
Secondary/tertiary	0.57	0.27	0.11	2.06	0.04	0.02	1.12
Economic status							
Poor	ref	ref	ref	ref	ref	ref	ref
Good	1.43	0.57	0.14	2.49	0.013	0.30	2.56
Outdoor leisure activity							
Yes	ref	ref	ref	ref	ref	ref	ref
No	0.70	0.33	0.11	2.11	0.036	0.04	1.35
Duration of residence							
< 2 years	ref	ref	ref	ref	ref	ref	ref
≥ 2 years	0.42	0.28	0.08	1.50	0.134	-0.13	0.97
Type of accommodation							
Ward	ref	ref	ref	ref	ref	ref	ref
Twin-sharing	-0.25	0.47	-0.03	-0.53	0.592	-1.18	0.67
Chronic co-morbidities							
Yes	ref	ref	ref	ref	ref	ref	ref
No	0.77	0.38	0.12	2.02	0.045	0.01	1.53
Social support							
Low	ref	ref	ref	ref	ref	ref	ref
High/medium	2.36	0.29	0.47	7.93	<0.0001	1.77	2.95

Multiple linear regression was used for analysis and *p*-value <0.05 was considered statistically significant

**Table 6:** Factors associated with Social Domain of QOL in Multivariate Analysis

Variable	B	S.E.	Beta	t	*P*-value	95 % C.I	
						Lower	Upper
Gender							
Female	ref	ref	ref	ref	ref	ref	ref
Male	0.79	0.38	0.11	2.07	0.039	0.04	1.55
Level of education							
None/primary	ref	ref	ref	ref	ref	ref	ref
Secondary/tertiary	0.80	0.36	0.11	2.18	0.030	0.07	1.52
Economic status							
Poor	ref	ref	ref	ref	ref	ref	ref
Good	1.23	0.74	0.08	1.65	0.100	-0.24	2.70
Outdoor leisure activity							
Yes	ref	ref	ref	ref	ref	ref	ref
No	1.11	0.40	0.13	2.53	0.012	0.24	1.97
Physical activity							
Yes	ref	ref	ref	ref	ref	ref	ref
No	0.34	0.40	0.04	0.87	0.385	-0.44	1.13
Duration of residence							
< 2 years	ref	ref	ref	ref	ref	ref	ref
≥ 2 years	1.45	0.36	0.36	3.93	<0.0001	0.72	2.17
Chronic co-morbidities							
Yes	ref	ref	ref	ref	ref	ref	ref
No	1.34	0.52	0.52	2.56	0.011	0.31	2.37
Social support							
Low	ref	ref	ref	ref	ref	ref	ref
High/medium	2.99	0.38	0.38	7.74	<0.0001	2.23	3.75

Multiple linear regression was used for analysis and *p*-value <0.05 was considered statistically significant

**Table 7:** Factors associated with Environment Domain of QOL in Multivariate Analysis

Variable	B	S.E.	Beta	t	*P*-value	95 % C.I	
						Lower	Upper
Gender							
Female	ref	ref	ref	ref	ref	ref	ref
Male	0.41	0.31	0.07	1.30	0.195	-0.21	1.04
Outdoor leisure activity							
Yes	ref	ref	ref	ref	ref	ref	ref
No	0.48	0.36	0.08	1.33	0.185	-0.23	1.20
Physical activity							
Yes	ref	ref	ref	ref	ref	ref	ref
No	0.44	0.33	0.08	1.32	0.188	-0.21	1.11
Duration of residence							
< 2 years	ref	ref	ref	ref	ref	ref	ref
≥ 2 years	0.53	0.31	0.10	1.74	0.083	0.07	1.15
Type of accommodation							
Ward	ref	ref	ref	ref	ref	ref	ref
Twin-sharing	0.86	0.51	0.10	1.66	0.097	0.15	1.88
Chronic co-morbidities							
Yes	ref	ref	ref	ref	ref	ref	ref
No	0.87	0.42	0.12	2.02	0.044	0.02	1.71
Social support							
Low	ref	ref	ref	ref	ref	ref	ref
High/medium	2.18	0.33	0.42	6.60	<0.0001	1.53	2.84

Multiple linear regression was used for analysis and *p*-value <0.05 was considered statistically significant

The psychological domain was significantly associated with gender (*p* = 0.003), level of education (*p* = 0.04), economic status (*p* = 0.013), outdoor leisure activity (*p* = 0.036), chronic co-morbidities (*p* = 0.045) and social support (*p* = <0.0001).

The social domain was significantly associated with gender (*p* = 0.039), level of education (*p* = 0.030), outdoor leisure activity (*p* = 0.012), duration of residence (*p* = <0.0001), chronic co-morbidities (*p* = 0.011) and social support (*p* = <0.0001). Chronic co-morbidities (*p* = 0.044) and social support (*p* = <0.0001) were significantly associated with the environment domain.

## Discussion

The physical domain of quality of life had the highest mean score 14.3 (±2.7) in this study, while the social domain had the lowest mean score 10.8 (±3.4). This was anticipated as a basic criteria for admission into these homes is the capacity to perform activities of daily living. In addition residents are usually abandoned by their relatives, and this explains the low scores in the social domain. Kumar et al. [28], in a study in India also reported lowest score in the social domain. This could be as a result of the growing number of elderly that face abandonment and neglect in India [29]. However, other studies [19, 30] have reported lower scores in the physical domain compared to other domains. This is because these studies were conducted in nursing homes, and such homes usually admit people with varying degrees of impaired physical function.

Age was only significantly associated with the physical domain. This is because the older age group had more functional limitations compared to the younger age group, a study by Tajvar et al. [19] reported impaired physical health among older age groups. As people age, the probability of developing physical health problems like musculoskeletal problems tends to increase. Women had a significantly lower quality of life in all domains compared to men. This could be because the women in these homes perceive aging more negatively than the men. Other studies [30, 31] reported lower quality of life scores among women and attributed their findings to feelings of unattractiveness among elderly women, which could lead to low self-esteem and also contribute to negative perception of ageing among elderly women. Marital status was not significantly associated with quality of life in this study. This is perhaps because married residents live in these homes without their spouses. Spouses only visit occasionally, and this could diminish the impact of the support that can be provided by a spouse. Previous studies [32, 33] reported similar findings. These studies were conducted in nursing homes, thus it is very likely that the married participants were not living with their spouses in the nursing homes and this could reduce the support that the spouses can provide.

Level of education was significantly associated with the physical, social and psychological domains of quality of life. Evidence from studies suggests that people with higher level of education are more likely to engage in healthy behaviors which could improve physical health compared to those with lower level of education [34]. In addition higher level of education can improve psychological resilience, coping mechanisms [35] and social relationships [36]. This explains the better quality of life among residents with higher level of education, as such residents are more likely to better manage stressors faced in these homes. Although there is little residents of these homes can do regarding the nature of their environment, those with higher level of education also had higher scores in the environment domain of quality of life. Previous studies [28, 37] have reported significantly better quality of life among people with higher level of education compared to those with lower level or no education, further highlighting the positive impact of higher education on quality of life.

Pension was not significantly associated with quality of life in this study. This is because the pension received by the residents might be inadequate to meet their basic needs, placing them in a comparable income class as those not receiving pension. This finding was not consistent with findings from previous studies [28, 38] that have reported a link between pension and quality of life. This is probably because the studies were conducted among community-dwelling elderly who could have additional source of income other than pension, and a better income could positively affect quality of life. Economic status was significantly associated with all domains of quality of life except the environment domain. This is perhaps because those with a good economic status may not be completely dependent on the elderly homes to provide needed services. This can have a positive impact on their physical and psychological health. A study by Tajvar et al. [19] reported a significant association between economic status and higher quality of life scores. The authors explained that better economic status was necessary to meet basic needs of life, participate in society and make elderly worry less about unexpected future expenses. All these can have a positive impact on quality of life.

Outdoor leisure activity was significantly associated with higher quality of life scores in all domains. This is because participation in leisure activities enhance good psychological feelings and physical functions [39]. In addition those that participate in such activities are more likely to have more social contacts with the outside community which could provide an additional source of support. Previous studies [32, 40] have reported an association between leisure activity and quality of life. These studies found out that leisure activities like trips into town, walking and gardening are very important factors that can improve quality of life among the elderly.

Smoking status was not significantly associated with quality of life in this study. However others studies [41, 42] have reported a significant association between quality of life and smoking status. This is because of the detrimental effect of smoking; its association with multiple non-communicable diseases which could negatively affect quality of life. Alcohol status was not significantly associated with quality of life in this study. This is probably because this study did not distinguish between moderate drinkers and heavy drinkers. Previous studies [41, 43] have reported significantly better quality of life among moderate drinkers when compared to non-drinkers. It has been reported that moderate consumption of alcohol is protective against heart diseases [44], and a vital social activity for the elderly [45].

Those that had lived in an elderly home for more than two years had higher quality of life scores in all domains compared to those that had lived for less than two years. The early months in the elderly home could be characterized by negative feelings such as worthlessness, rejection, loneliness, and insecurities following abandonment. Over time, as residents adjust to their new environment, they make new friends, and possibly develop a positive attitude. Tseng and Wang in a study in Southern Taiwan [33], reported a significant negative correlation between quality of life and duration of residence. This is because their study was conducted among nursing home residents with varying degrees of physical dependence and poor health, thus those with worse health conditions are more likely to live in the homes for prolonged periods.

Those living in twin-sharing accommodations had significantly higher quality of life in the environment and psychological domains compared to those living in ward accommodations. Twin-sharing accommodations provided social and psychological privacy, while those in ward-type accommodations barely have visual privacy. Furthermore twin-sharing accommodations provide ample space, better opportunities for residents to personalize their rooms to suit themselves, and better appreciate their environment, all of which could improve sense of identity and overall mental health and quality of life. Cooney et al. [46], in a study conducted in Ireland also reported better quality of life among residents living in more private accommodation than those living in ward-type accommodation. The authors identified lack of space, privacy, peace and quiet as the common problems faced by those living in ward-type accommodations.

Those with a chronic co-morbidity had significantly lower quality of life scores in all domains. In the absence of proper medical care in these homes, residents with chronic co-morbidities are likely going to bear the brunt of the different health conditions they have, and this could negatively affect them both physically and emotionally. Similar findings were reported in other studies [40, 41]. Chronic conditions usually require constant medical attention and lifestyle modifications, and as the number of co-morbidities increases there could also be increase in functional impairment, frequent hospitalization, adverse drug effects, and mortality.

Those with higher level of social support had significantly higher quality of life scores in all domains. Those with higher levels of social support are least likely to feel abandoned because they still have people they can count on. In addition, higher levels of social support could lead to reduced risk of mental disorders, physical disease, mortality and improved quality of life [12–14]. These findings were consistent with previous studies [33, 10], that also reported that social support is crucial for the elderly, it makes them feel loved, valued and prevent them from feeling abandoned. The findings of this study provides an insight on the quality of life of residents of these homes, it also highlights the range of factors that affect it. The neglect of residents of these homes takes quite a toll on their quality of life. These findings could guide interventions aimed at improving the health and overall quality of life of the elderly in elderly homes. There is need for multifactorial active ageing interventions to improve the quality of life in these homes, particularly the social component. These interventions should include administrators of these homes, the residents themselves, families and even policy makers in Malaysia.

This study is not without limitations. It is a cross sectional study, thus a temporal relationship between quality of life and the associated variables cannot be established. In addition the study participants were not nationally representative, as only elderly homes in Kuala Lumpur were included. Thus the findings cannot be extrapolated to the entire country. This study measured perceived social support, and this does not indicate the actual resources or support received by participants. Cohort studies are recommended in this area to determine causal-relationship between quality of life and associated factors. These studies should include elderly homes in different states in Malaysia to allow extrapolation of findings. Other studies should go beyond measuring perceived social support by measuring real support, and the type of support most needed by elderly in elderly homes.

## Conclusion

The findings of this study indicate that residents of these elderly homes had worse quality of life scores in the social domain and the best in the physical domain. Age, gender, level of education, economic status, outdoor leisure activity, physical activity, duration of residence, type of accommodation, chronic co-morbidities, and social support were significantly associated with at least one domain of quality of life. These findings accentuates the need for active ageing interventions to ameliorate the quality of life of residents of these homes.
